# Frequency, Types, and Manifestations of Partner Sexual Violence, Non-Partner Sexual Violence and Sexual Harassment: A Population Study in Spain

**DOI:** 10.3390/ijerph19138108

**Published:** 2022-07-01

**Authors:** Guadalupe Pastor-Moreno, Isabel Ruiz-Pérez, Luis Sordo, Jesús Henares-Montiel

**Affiliations:** 1 Escuela Andaluza de Salud Pública, 18011 Granada, Spain; guadalupe.pastor.easp@juntadeandalucia.es (G.P.-M.); jesus.henares.easp@juntadeandalucia.es (J.H.-M.); 2 Consorcio de Investigación Biomédica y en Red de Epidemiología y Salud Pública (CIBERESP), 28029 Madrid, Spain; lsordo@ucm.es; 3 Instituto de Investigación Biosanitaria de Granada (ibs. GRANADA), 18012 Granada, Spain; 4 Departamento de Salud Pública y Materno-Infantil, Facultad de Medicina, Universidad Complutense de Madrid, 28040 Madrid, Spain

**Keywords:** sexual violence, intimate partner violence, sexual harassment, cross sectional survey, complaint, Spain

## Abstract

Background: This study analyzes the frequency and sociodemographic characteristics associated with sexual violence by a partner/ex-partner (PSV), someone other than a partner or ex-partner (NPSV), or sexual harassment (SH). Methods: The study is based on the 2019 Macro-survey of Violence against Women conducted by the Spanish Ministry of Equality among a sample of 9568 women age 16 or more years. Odds Ratios were calculated and multinomial logistic regression analyses were performed. Results: Forty-four women had suffered some kind of sexual violence over their lifetime, 9.2% had experienced PSV, 6.5% NPSV and 40.4% SH. More than 7% of women had been raped by a partner and 2.2% by another man. In the three groups, violence was associated with lower age and having a certified disability. NPSV and SH were significantly associated with a higher education and internet use. In NPSV, 9.2% of cases were reported to the police and 3.9% were reported to the courts. In SH, 91.7% of women told a family member or a close friend and 4.2% reported it to the police or the courts. Conclusions: A greater emphasis needs to be placed on reporting sexual violence in its various forms. Rape within intimate partnerships ought to be investigated and studied in greater depth.

## 1. Introduction

Sexual violence comprises “any sexual act, attempt to obtain a sexual act, unwanted sexual comments or advances, or acts to traffic or otherwise directed against a person’s sexuality using coercion, by any person regardless of their relationship to the victim, in any setting, including but not limited to home and work” [1].

### 1.1. Key Definitions and Limitations

The term sexual violence in scientific literature ranges from acts that involve socially disapproved actions (such as child abuse or rape) to other normalized behaviors in the social sphere (such as sexual harassment in the street or the workplace). In general, the first differentiation is according to the perpetrator of the violence [2].

Sexual violence perpetrated by a partner is referred to as Partner Sexual Violence (PSV) and has often been grouped into perpetrations of intimate partner violence (IPV). Sexual violence perpetrated by those such as strangers, acquaintances, friends, colleagues, peers, teachers, neighbors, and family members is referred to as non-partner sexual violence (NPSV) [3].

PSV has been addressed less frequently than NPSV in the scientific literature [4,5]. It is common in studies addressing IPV to ask about the frequency of sexual violence, together with physical violence [6,7,8]. Because PSV lies at the intersection of IPV and sexual violence, it is often overlooked, and research on PSV lags behind research on other forms of violence against women [4]. This has made PSV more difficult to detect and it remains underreported [9,10,11].

In addition, the variability of definitions and populations studied mean that outcomes are not always comparable and that we find high variability in frequencies of PSV and NPSV depending on the geographical setting of the studies.

Sexual Harassment (SH) as defined by the World Health Organization WHO [12] is any unwelcome sexual behavior or request for sexual favors. This includes verbal or physical conduct, or any gesture of a sexual nature, particularly behavior construed as offensive or intimidating to the person concerned. Sexual harassment is one of the forms of sexual violence that has traditionally been most studied [13]. This interest has been boosted in recent years motivated, in part, by the media impact and social repercussions of specific acts, such as the accounts of sexual harassment given by victims themselves arising from viral phenomena such as #MeToo [14,15].

### 1.2. Frequencies of Different Forms of Sexual Violence

According to WHO estimates for 2018 [16], globally, 27% of ever-married/partnered women of reproductive age (15–49 years) are estimated to have experienced physical and/or sexual IPV at least once in their lifetime. PSV figures could be higher than 50% in some populations [5], although data from population surveys show that the lifetime prevalence of PSV varies between 3.3% and 18.3% [17,18,19].

Regarding NPSV, globally, 6% of women aged 15–49 years report that they have been subjected to NPSV at least once in their lifetime [16]. The highest estimated prevalence of NPSV is in high-income regions including Australia and New Zealand (19%) and Northern America (15%). Substantial variation in prevalence was seen across regions in Northern Europe (10%) and in Asia (3%). Results from population surveys estimate a prevalence of NPSV of between 1.7% and 43.6% [3,17,18,19].

As for the frequency of SH, a study conducted on sexual harassment and assault [20] shows that verbal sexual harassment is most common, as reported by 77% of women, and physically aggressive forms of sexual harassment were reported by 62% of women. It is estimated that between 45% and 55% of European women and 37% of American women have suffered some type of SH throughout their lives [18,19].

### 1.3. Sociodemographic Characteristics Associated with Different Forms of Sexual Violence

Different studies show that women sexually assaulted by a current or former intimate partner differed from other sexual assault survivors in some socio-demographic characteristics [17,21].

In the case of PSV, a higher frequency of this form of violence has been found in women with a lower socioeconomic level, women with a greater number of children, living with more people in the household, of younger age and lower educational level [22,23,24].

Regarding NPSV, in the US some studies show that victims are more often women from ethnic minorities and young [25,26], although a recent longitudinal study found no significant association between ethnicity or age and sexual violence [27].

Respecting SH, it is a common problem among women of all origins, although the factors associated with a higher frequency of SH depend on the setting of the study (college, school, workplace, home, online space, hospital and army). Thus, for example, studies carried out in the academic context (college and university) have identified belonging to a sexual minority and being white as risk factors [28,29], while a recent study of SH in public spaces in the UK indicates that the prevalence is higher among younger women [30].

### 1.4. Formal and Informal Reporting of Sexual Violence

One of the ways out of a situation of sexual violence is to file a complaint with the authorities. However, according to the WHO, less than 40% of women who experience violence in general seek some kind of help and most do so from family members and friends. Very few turn to formal institutions, such as the police or health services. Fewer than 10% of those that seek help go to the police [31].

Furthermore, official records provide an approximation only of the number of women who file a complaint, depending on the definitions used, and therefore do not reflect the reality of the problem. For example, the legal conditions as regards rape statistics differ between countries and European countries differ in terms of the sexual acts included under rape in their national legislation. The propensity of victims to report rape is of considerable importance regarding the number of reported crimes in the statistics [32].

### 1.5. Current Study

In Spain, the Macro-survey of Violence against Women is the only official statistical operation conducted to measure the prevalence of gender-based violence [33]. The survey is conducted every four years and the objectives are to estimate the prevalence and types of violence against women, differentiating between partners/ex-partners and outside parties, and to analyze the effects on the health and work of victims, and the formal and informal support they receive.

This survey meets the quality requirements for statistics on violence against women recommended by the United Nations Statistical Division [34] and the design of the questions is based on the recommendations of the Istanbul Convention [35]. The questionnaire is exhaustive when it comes to collecting the different dimensions of violence against women and allows us to delve into different forms and types of violence.

The report published by the Government of Spain offers an overview of the frequencies of different types of violence. However, it does not carry out statistical analysis that specifically delves into the associated factors for each form of sexual violence. For this reason, the Spanish government makes the survey database available to the scientific community, to facilitate the performance of specific analysis [36,37,38].

The opportunity of having a population-based survey with a large sample that studies independently, in the same population, the different forms of violence that a woman may experience throughout her life (PSV, NPSV and SH) allows us to set up the following objectives: (a) to study the number of women who have experienced any form of sexual violence throughout their lives; (b) to study the types and frequency and the sociodemographic characteristics associated with PSV and NPSV; (c) to analyze the relationship between the frequency of PSV and NPSV and the filing of complaints; (d) to study the frequency and types of SH and to analyze the sociodemographic characteristics associated with SH; (e) to analyze the relationship between the frequency of SH and the disclosure of such harassment to other people or institutions.

## 2. Materials and Methods

### 2.1. Study Design and Population

This study is based on the 2019 Macro-survey of Violence against Women conducted by the Spanish Ministry of Equality on a sample of 9568 women over the age of 16 years resident in Spain [33]. The survey was carried out by the Sociological Research Center (a public organization whose purpose is the scientific study of Spanish society) using multistage stratified cluster sampling, with proportionate random selection of the primary sampling units (municipalities) and secondary units (sections) and random-route selection of the final units (individuals) with age and occupation quotas.

The information was collected between September and December 2019 through computer-assisted personal interviews (CAPI) at women’s homes and using response cards for the most sensitive questions to facilitate privacy. The women participated voluntarily and did not receive any remuneration. The information on the variables and frequency distributions of the Macro-survey can be consulted at the following website: http://www.cis.es/cis/export/sites/default/-Archivos/Marginales/3220_3239/3235/es3235mar.html (accessed on 5 September 2021).

### 2.2. Variables

For this study we selected three variables with respect to self-reported sexual violence:

(a) Partner or ex-partner Sexual Violence (PSV).
(b) Non-Partner Sexual Violence (NPSV).

PSV and NPSV were measured with 8 items, on the basis of which two forms of violence can be distinguished: rape (with the first four items, which are those that include situations related to forcing women to have sexual relations) and sexual abuse (with the last four items) (Appendix A). A woman was considered to have experienced PSV or NPSV if she answered affirmatively to one of the 8 items.

(c) Sexual Harassment (SH).

SH was measured with 11 items (Appendix A). To screen women who had experienced this form of violence, they were asked whether they had experienced any unwanted behaviors with a sexual connotation from any person. A woman was considered to have experienced SH if she answered affirmatively to one of the items.

These three variables are not mutually exclusive and women had to respond indicating whether they had experienced that situation in their lifetime.

NPSV and SH respondents were asked about the nature of their relationship to the perpetrator (family member; friend or neighbor; someone from work or class; person belonging to a religious institution; person known by sight or unknown).

PSV and NPSV respondents were asked whether they had filed a judicial complaint, and whether they had reported the assault to the police. If they responded affirmatively, they were asked who had filed the complaint or reported the assault to the police (themselves/other people or institutions). The women who had not done so themselves were asked their reasons for not doing so (answer options shown on card: She solved it alone/ended the relationship; She was a minor; Not considering the matter important/not knowing that she could file a complaint; She was afraid of the aggressor/Someone dissuaded or prevented her; Out of shame, feelings of guilt, or fear of not being believed; The problem is over; She lacked her own financial resources/went elsewhere to ask for help; She did not want to lose her partner or have her children lose their father; They were other times/it happened in another country).

SH respondents were asked whether they had spoken to anyone or to any institution about the sexual harassment they had experienced.

The following sociodemographic variables were included, based on available scientific research [11,39]: age (16–25/26–45/46–65/≥66); size of municipality (0–10,000/10,001–100,000/≥100,001); educational attainment (Primary or less/Secondary (high school)/Higher (university)); household size (1/2/≥3); having children (no/yes); country of birth (Spain/Other); employment situation (Unemployed or Housewife/Employed/Retired/Student); level of family income (≤€300 per month/€301–900 per month/€901–1800 per month/€1801–3000 per month/>€3000 per month), Internet use (no/yes); degree of disability of 33% or more (no/yes); religiosity (no/yes); having a trusted person (no/yes).

### 2.3. Statistical Analysis

For descriptive univariate analyses, we used frequency tables for categorical variables and means, medians, standard deviations, minima, and maxima for continuous variables. The prevalence of PSV, NPSV, and SH was calculated for lifetime. For the bivariate logistic analysis and raw odds ratios (ORs), the chi-square test was used to analyze the relationships between categorical variables, and Student’s *t*-test was used to analyze the relationships between categorical and continuous variables. Adjusted ORs were calculated and multinomial logistic regression analyses were performed in order to identify the factors that were associated with each violence category. Statistical significance was set at *p* < 0.05. Throughout the analysis we used the weightings included in the 2019 Macro-survey of Violence Against Women. The analyses were performed using R statistical software.

## 3. Results

**Figure 1:**
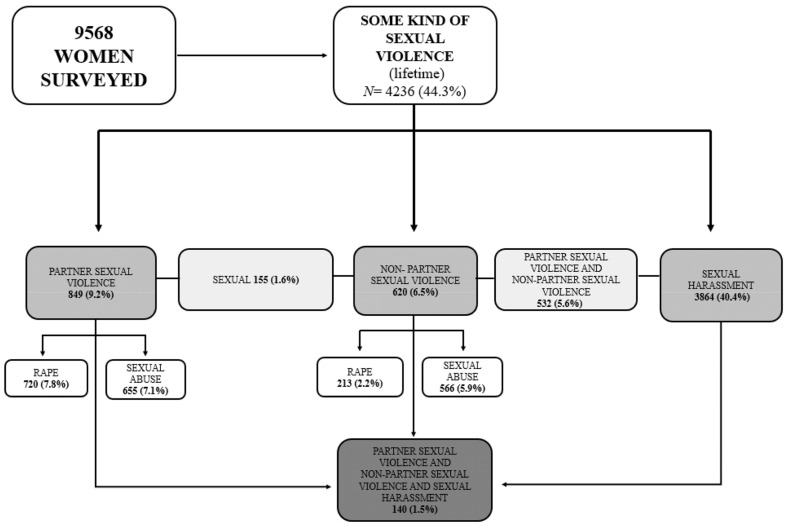
Frequency and combinations of sexual violence: Partner Sexual Violence, Non-Partner Sexual Violence and Sexual Harassment.

Forty four percent of women had experienced some kind of sexual violence in the course of their lives (PSV, NPSV, or SH). Taking into account the accumulation of types of sexual violence analyzed, 1.5% of the women had experienced all three at some point (Figure 1).

### 3.1. Partner Sexual Violence and Non-Partner Sexual Violence

#### 3.1.1. Frequency

Of the surveyed women, 9.2% of women had experienced PSV (7.8% rape and 7.1% sexual abuse) and 6.5% NPSV (2.2% rape and 5.9% sexual abuse). The perpetrator of PSV in 98.4% of cases was a man. A total of 60.3% of the women indicated that it was a man they knew by sight or a stranger, 23.2% a family member, 22.4% a friend or neighbor, 11.6% a fellow worker or student, and 0.32% a member of a religious institution.

#### 3.1.2. Characteristics of Women Associated with Partner Sexual Violence and Non-Partner Sexual Violence

Table 1 and Table 2 shows the association between PSV and NPSV and various sociodemographic, occupational, and social support characteristics of the women, as well as according to recognized disability.

In the adjusted analysis (Table 1), PSV was significantly associated with: lower age (*aOR* = 3.44, 95% CI [1.86, 6.29]) for women younger than 25 versus those older than 66; having children (*aOR* = 1.80, 95% CI [1.36, 2.41]); being employed (*aOR* = 1.45, 95% CI [1.15, 1.84]) versus unemployed women and housewives; and lower family income level (*aOR* = 2.70, 95% CI [1.86, 3.99]) among women with incomes between 301 and 900 euros per month versus those with incomes of more than 3000 euros per month. The prevalence was lower among women living in two-person households (*aOR* = 0.69, 95% CI [0.52, 0.91]). Finally, women with a certified disability were more likely to experience PSV (*aOR* = 2.34, 95% CI [1.70, 3.16]). No statistically significant relationships were found for the other variables.

NPSV (Table 2) was significantly associated with lower age (*aOR* = 2.85, 95% CI [1.69, 4.87]), higher education (*aOR* = 2.31, 95% CI [1.53, 3.56]), Internet use (*aOR* = 2.24, 95% CI [1.36, 3.80]), and certified disability (*aOR* = 1.99, 95% CI [1.35, 2.86]). Living in households of three or more persons was associated with a lower prevalence of PSV (*aOR* = 0.64, 95% CI [0.46, 0.90]). No statistically significant relationships were found for the other variables.

#### 3.1.3. Prevalence for Filing Police Reports or Complaint and Reasons for Not Filing

Of the women who had experienced some kind of physical, sexual, or emotional partner violence lifetime, 21.7% had reported it to the police or filed a complaint with the courts. Of these, 54.3% had experienced PSV (Not shown in Figure 1).

**Figure 2:**
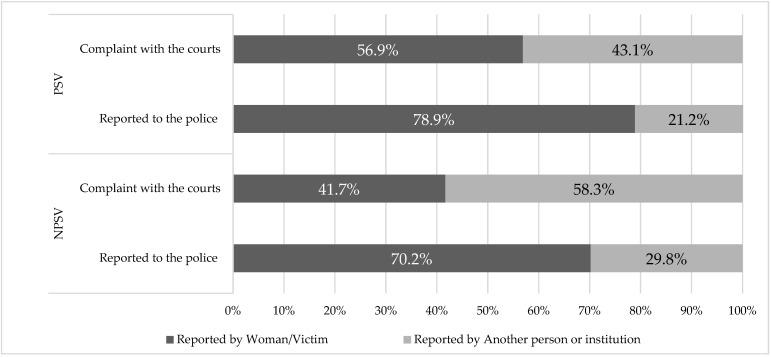
Person who files the complaint and reports PSV and NPSV to the police.

In 78.9% of cases it was the woman herself who reported the assault to the police and in 21.2% it was another person or institution. In the case of judicial complaints, in 56.9% of cases it was the woman herself who filed suit against her partner or ex-partner and in 43.1% it was another person or institution (Figure 2, PSV).

With regard to NPSV, 9.2% of cases were reported to the police either by the woman herself (70.2%) or by other people or institutions (29.8%). In the case of judicial complaints, suit was filed in 3.9% of cases, either by the woman herself (41.7%) or by other people or institutions (58.3%) (Figure 2, NPSV).

**Figure 3:**
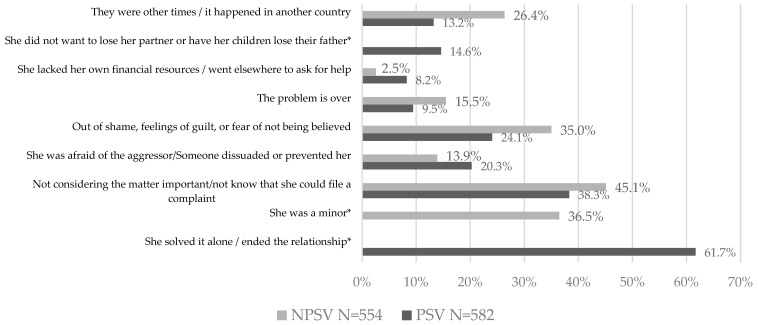
Reasons for not filing a police report or judicial complaint of PSV and NPSV. * Some answers do not apply for a certain group of women, so the data is not shown.

In the case of PSV, the most commonly cited reasons for not filing a complaint were having resolved the issue alone or put an end to the relationship (61.7%), not considering the violence experienced important or not knowing that a complaint could be filed (38.3%), and feeling ashamed, guilty, or afraid of not being believed (24.1%). As for NPSV, the reasons most frequently given were not considering the matter important or not knowing that a complaint could be made (45.1%), being a minor when the violence occurred (36.5%), and in third place feeling ashamed, guilty, or afraid of not being believed (35%) (Figure 3).

### 3.2. Sexual Harassment

#### 3.2.1. Frequency and Types of SH

The various reported behaviors corresponding to SH were grouped as shown in Appendix A.

Out of all the women who had experienced SH in their lifetime (40.4%, *N* = 3864), 43.4% had been subjected to inappropriate physical contact, 40.5% to exhibitionism, and 34.7% to intimidation. The same proportion, 34.7%, had received inappropriate suggestions about going on a date or engaging in some activity of a sexual nature, 25.1% had received inappropriate content, 5.3% sexual harassment in the workplace, and 9.4% other similar behavior with sexual connotations that had made them feel offended, humiliated, or intimidated.

With respect to their relationship to the harasser, in 92.9% of cases the perpetrators had been exclusively men, in 0.7% women, and in 5.3% both men and women. In the case of male perpetrators, 93.9% of the women indicated that they knew them by sight or they were strangers, 26.4% said that they were a workmate or fellow student, 15% a friend or neighbor, 7.4% a family member, and 0.4% a member of a religious institution. In the case of women harassers, 3.8% of the women indicated that they knew the perpetrator by sight or she was a stranger, 2.1% said that she was someone from work or class, 1.6% a friend or neighbor, and 0.3% a family member.

#### 3.2.2. Sociodemographic Characteristics Associated with SH

In the adjusted analysis (Table 3), a greater prevalence of SH was significantly associated with being younger (*aOR* = 3.18, 95% CI [2.24, 4.54] for women under 24 versus those over 66, living in municipalities with more than 100,000 inhabitants (*aOR* = 1.35, 95% CI [1.15, 1.57]), having completed secondary education (*aOR* = 1.85, 95% CI [1.54, 2.24]) and higher education (*aOR* = 2.84, 95% CI [2.28, 3.54]), being a student (*aOR* = 2.44, 95% CI [1.64, 3.66]), and using the Internet (*aOR* = 1.91, 95% CI [1.53, 2.38]). The prevalence of SH was lower in women from households with three or more members (*aOR* = 0.77, 95% CI [0.64, 0.94]). Women with a certified disability were more like to experienced SH (*aOR* = 1.64, 95% CI [1.29, 2.07]).

#### 3.2.3. Disclosure to Other People and Institutions

Of the 3864 women who stated that they had been sexually harassed, 39.6% did not speak to anyone about the subject and 60.1% sought help or spoke about it to some person or institution. Among the women who did tell someone about their SH, 91.7% told a family member or close friend and 14.3% a fellow worker or student. Only 4.2% of women reported it to the police or the courts and 3.4% consulted some professional service (such as medical, psychological, social, or legal services). Finally, 1% reported it on social media (Table 4).

#### 3.2.4. Characteristics of Women Who Do Not Talk about SH

Table 5 shows the sociodemographic characteristics of women who did not talk about the SH they had experienced versus those who did do so. The adjusted analysis indicates that the probability of not speaking about it was significantly higher among women over the age of 66 (*aOR* = 2.34, 95% CI [1.55, 3.55]), those born outside Spain (*aOR* = 1.46, 95% CI [1.07, 1.99]), those who did not use the Internet (*aOR* = 1.60, 95% CI [1.12, 2.31]), those who did not have a trusted person to speak to (*aOR* = 1.61, 95% CI [1.12, 2.33]), and those who considered themselves religious (*aOR* = 1.26, 95% CI [1.04, 1.51]). No statistically significant relationships were found for the other variables.

## 4. Discussion

In this study, we address the sexual violence that occurs not just in partnerships but also outside of them and describe the characteristics of women who report it and the reasons for not doing so. We also analyze various aspects of SH, such as frequency, types, characteristics of women who are harassed, and seeking help. In addition, we have addressed rape in the context of intimate partnerships, a subject that has so far received little attention [26].

One of the points to be highlighted is that in Spain more women have been raped by a partner during their lifetime (7.8%) than by other persons (2.2%). In the United States, it is estimated that 45.4% of women who have experienced rape have been raped during their lifetimes by a current/former intimate partner, 12.1% by a family member, and 12.9% by a stranger [26]. One of the most pernicious myths about rape is that it is an act committed by a stranger [21,40], and the stigma that characterizes domestic abuse as a “private matter” rather than criminal behavior that should receive the attention of law enforcement.

As for the perpetrators, we must highlight the fact that, in the case of NPSV, almost half the perpetrators are men from the victim’s immediate circle. Previous studies and reports have indicated that only between 18.6% and 22.0% of rapes are committed by strangers [41,42] and that most assaults occur in the victim’s or the perpetrator’s home, which confirms that perpetrators are people the victim knows [18]. This should lead us to reconsider the myth of the attacker in a dark alley and help us to understand, in part, the low number of complaints for NPSV.

In our study, younger and employed women were at higher risk of experiencing PSV. In studies that analyze partner violence in general, the groups at highest risk are retired and unemployed women [8], which shows that when sexual violence is studied separately from other kinds of violence, more specific profiles of women emerge [43]. Furthermore, having children and having lower income were also associated with PSV, which seems to indicate that women who experienced PSV have more difficulties in being emotionally and economically independent from the male partner, and, therefore, to abandon the abusive relationship [44].

Regarding women who experienced NPSV, they were younger, from smaller households, with higher education level, and users of the Internet. Perhaps it is these groups of women that are best informed, and therefore more socially aware and less tolerant of behavior that may be normalized and not regarded as sexual violence, since the Internet and social media is a window onto greater information and knowledge about the realities of other women [45].

It can be seen that women with disabilities are more vulnerable to experiencing PSV, NPSV and SH, as the previous literature indicates [26,46]. This situation is a wake-up call to disability evaluation services and also merits an in-depth approach that will help us to understand what other factors are involved in the association between sexual violence and disability, as has been done with other groups of women.

Despite all the data, the number of women who report sexual violence to the authorities is very low, both for PSV and NPSV. In Europe, it is estimated that only 20% of affected women report having experienced physical or sexual violence [18]. If, despite the fact that there are more channels of information, filing a judicial complaint or a report to the police is not one of the main solutions when faced with a situation as traumatic as sexual violence, we should ask ourselves whether the help mechanisms and services are working effectively in the fight against gender-based violence. One of the strengths of this study is that it enables us to discover the motivations of women who do not file a complaint. In a previous study in Canada [47], the most common reasons provided were that the crime was minor and it was not worth taking the time to report it (71%), that the incident was a private or personal matter and it was handled informally (67%), and that no one was harmed during the incident (63%).

Our results are striking in two senses: the high percentage of women that responded who did not report the NPSV because they did not know that a complaint could be made, and on the other hand the high percentage of women who stated that they had solved it alone. The latter point raises the suspicion that the women who responded in this way had not in practice done anything to resolve the situation.

This study is one of the first to reveal the frequency of SH through a population survey, which is self-reported by two out of five women, which highlights the magnitude of this social problem, often minimized compared to other forms of sexual violence. Women who report that they have experienced SH are those with a college student-like profile: young, living in urban areas, high education attainment, user of the Internet and low income. Perhaps, as stated before, the women in this group have greater social awareness when it comes to identifying and verbalizing SH. However, it could also be that they are more exposed to strangers, who in this case are the most frequent perpetrators [30].

The women who reported SH most commonly turned to someone very close to them to talk about it and only 4% filed a complaint. This shows that if we turn to police records to analyze the problem we will probably obtain an approximation that underestimates the magnitude of the problem. Moreover, only 1% of women who did tell someone about it did so on social media, despite the fact that the profile could correspond to women who use social media. Perhaps the #MeToo phenomenon has not taken root sufficiently [14].

Of the women who responded, 44.5% had experienced some kind of violence (PSV, NPSV or SH) and 1.5% had experienced all three of the kinds analyzed here. Bearing in mind the serious impact of sexual violence on health [5,48], this form of violence, in any of its types, ought to be more present in research on gender-based violence.

The limitations of this study are those inherent in any cross-sectional study. Furthermore, this survey shows the number of women who acknowledge and are prepared to reveal the assault. Social desirability and the stigma that sexual violence still entails today may lead to these experiences not being declared, or perhaps the first condition to combat sexual violence and harassment is that the cases should be perceived as such, since research has shown that perception depends on factors such as context, ideology, and relationship to the perpetrator [49,50]. In any case, this would give rise to underestimation in the data obtained

## 5. Conclusions

This article reveals the magnitude of three distinct forms of sexual violence, PSV, NPSV, and SH, in a same sample population from Spain. Sexual violence has been described as one of the most degrading and humiliating experiences a person can endure and in this study it was reported by 44.3% of women. However, the percentage of criminal complaints is very low, outside as well as within intimate partnerships. Campaigns stressing the need to bring complaints are associated with physical violence and greater emphasis needs to be placed on reporting sexual violence in its various types, not only those that are receiving most attention in the media, such as sexual harassment, but also rape within intimate partnerships, which needs to be investigated further and studied in greater depth [51].

## Figures and Tables

**Table 1:** Association between sociodemographic characteristics and PSV.

	Sexual Violence in Partnerships (PSV) *N* = 849 (9.23%)		
	*n* (%)	OR [95% CI] *	*a*OR [95% CI] **
**Age**			
≥66	132 (6.16%)	1	1
46–65	**297 (9.51%)**	**1.56 [1.25–1.95]**	**1.67 [1.14–2.44]**
26–45	**311 (10.34%)**	**1.82 [1.47–2.27]**	**2.56 [1.66–3.96]**
16–25	**109 (11.76%)**	**2.10 [1.58–2.77]**	**3.44 [1.86–6.29]**
**Size of municipality**			
0–10,000	154 (8.65%)	1	
10,001–100,000	344 (9.56%)	1.10 [0.90–1.34]	
≥100,001	352 (9.21%)	1.13 [0.93–1.39]	
**Educational attainment**			
Primary or less	86 (8.89%)	1	
Secondary (high school)	320 (8.89%)	**1.36 [1.05–1.79]**	
Higher (university)	86 (5.98%)	0.94 [0.68-1.31]	
**Household size**			
1	132 (10.39%)	1	1
2	256 (8.28%)	**0.73 [0.58–0.91]**	**0.69 [0.52–0.91]**
≥3	457 (9.46%)	**0.79 [0.65–0.98]**	**0.52 [0.38–0.72]**
**Having children**			
No	216 (8.93%)	1	1
Yes	629 (9.70%)	1.02 [0.86–1.20]	**1.80 [1.36–2.41]**
**Country of birth**			
Spain	756 (8.91%)	1	
Other	93 (12.99%)	**1.52 [1.20–1.91]**	
**Employment situation**			
Unemployed/Housewife	222 (9.09%)	1	1
Employed	429 (10.17%)	**1.21 [1.02–1.44]**	**1.45 [1.15–1.84]**
Retired	152 (7.44%)	0.82 [0.65–1.02]	1.18 [0.84–1.68]
Student	42 (9.29%)	1.13 [0.78–1.60]	1.60 [0.83–2.98]
**Level of family income**			
>3000€/month	52 (7.17%)	1	1
1801–3000€/month	144 (8.05%)	1.10 [0.79–1.56]	1.08 [0.76–1.55]
901–1800€/month	238 (10.30%)	**1.50 [1.10–2.10]**	**1.70 [1.22–2.42]**
301–900€/month	159 (13.85%)	**2.15 [1.55–3.05]**	**2.70 [1.86–3.99]**
≤300€/month	14 (11.86%)	1.65 [0.91-2.87]	**2.03 [1.09–3.66]**
**Internet use**			
No	125 (6.88%)	1	1
Yes	719 (9.74%)	**1.45 [1.19–1.78]**	1.36 [1.00-1.87]
**Degree of disability ≥ 33%**			
No	770 (8.69%)	1	1
Yes	79 (1.34%)	**1.78 [1.38–2.28]**	**2.34 [1.70–3.16]**

* Statistically significant associations are indicated in bold. ** Only statistically significant associations are shown.

**Table 2:** Association between sociodemographic characteristics and NPSV.

	Sexual Violence Non–Partnerships (NPSV) *N* = 620 (6.48%)		
	*n* (%)	OR [95% CI] *	*a*OR [95% CI] **
**Age**			
≥66	56 (2.55%)	1	1
46–65	198 (6.21%)	**2.46 [1.82–3.39]**	1.50 [0.98–2.32]
26–45	249 (8.13%)	**3.19 [2.38–4.37]**	**1.86 [1.20–2.94]**
16–25	117 (10.48%)	**4.31 [3.08–6.10]**	**2.85 [1.69–4.87]**
**Size of municipality**			
0–10,000	105 (5.60%)	1	
10,001–100,000	287 (7.68%)	1.25 [0.98–1.60]	
≥100,001	229 (5.79%)	**1.45 [1.14–1.86]**	
**Educational attainment**			
Primary or less	35 (2.84%)	1	1
Secondary (high school)	238 (6.60%)	**2.55 [1.76–3.83]**	1.51 [1.02–2.28]
Higher (university)	115 (7.99%)	**3.05 [2.04–4.69]**	**2.31 [1.53–3.56]**
**Household size**			
1	85 (6.29%)	1	1
2	195 (6.20%)	1.04 [0.79–1.38]	0.78 [0.56–1.10]
≥3	337 (6.66%)	1.06 [0.82–1.39]	**0.64 [0.46–0.90]**
**Having children**			
No	257 (9.30%)	1	
Yes	363 (5.34%)	**0.54 [0.46–0.65]**	
**Country of birth**			
Spain	556 (6.30%)	1	
Other	64 (8.58%)	1.21 [0.90–1.60]	
**Employment situation**			
Unemployed/Housewife	158 (6.30%)	1	
Employed	329 (7.65%)	1.22 [1.00–1.50]	
Retired	79 (3.73%)	**0.57 [0.43–0.76]**	
Student	52 (8.78%)	**1.44 [1.01–2.01]**	
**Level of family income**			
>3000€/month	9 (7.20%)	1	
1801–3000€/month	76 (10.38%)	0.74 [0.55–1.01]	
901–1800€/month	136 (7.47%)	**0.53 [0.39–0.72]**	
301–900€/month	136 (5.72%)	**0.56 [0.39–0.80]**	
≤300€/month	71 (5.95%)	0.75 [0.39–1.35]	
**Internet use**			
No	31 (1.66%)	1	1
Yes	589 (7.65%)	**4.99 [3.47–7.48]**	**2.24 [1.36–3.80]**
**Degree of disability ≥ 33%**			
No	563 (90.81%)	1	1
Yes	57 (9.19%)	**1.77 [1.31–2.34]**	**1.99 [1.35–2.86]**

* Statistically significant associations are indicated in bold. ** Only statistically significant associations are shown.

**Table 3:** Association between sociodemographic characteristics and SH.

	*N* = 3864 *n* (%)	OR [95% CI] *	*a*OR [95% CI] **
**Age**			
≥66	672 (30.55%)	1	1
46–65	1502 (47.11%)	**2.29 [2.02–2.60]**	**1.43 [1.12–1.82]**
26–45	1209 (39.46%)	**3.50 [3.08–3.97]**	**1.97 [1.50–2.58]**
16–25	481 (43.10%)	**6.08 [5.18–7.16]**	**3.18 [2.22–4.54]**
**Size of municipality**			
0–10,000	667 (35.59%)	1	1
10,001–100,000	1731 (46.32%)	**1.34 [1.19–1.50]**	1.16 [1.00–1.35]
≥100,001	1466 (37.05%)	**1.54 [1.37–1.73]**	**1.35 [1.15–1.57]**
**Educational attainment**			
Primary or less	470 (20.29%)	1	1
Secondary (high school)	2185 (43.37%)	**3.13 [2.79–3.52]**	**1.85 [1.54–2.24]**
Higher (university)	1204 (54.50%)	**4.72 [4.13–5.40]**	**2.84 [2.28–3.54]**
**Household size**			
1	442 (32.72%)	1	1
2	1198 (38.07%)	**1.30 [1.13–1.49]**	1.03 [0.86–1.25]
≥3	2221 (43.89%)	**1.59 [1.40–1.82]**	**0.77 [0.64–0.94]**
**Having children**			
No	1487 (53.80%)	1	
Yes	2377 (34.94%)	**0.43 [0.39–0.47]**	
**Country of birth**			
Spain	3530 (40.01%)	1	
Other	334 (44.77%)	1.09 [0.94–1.26]	
**Employment situation**			
Unemployed/Housewife	880 (35.09%)	1	1
Employed	2006 (46.64%)	**1.55 [1.40–1.72]**	1.12 [0.97–1.29]
Retired	578 (27.32%)	**0.65 [0.58–0.74]**	1.14 [0.91–1.43]
Student	378 (63.85%)	**3.56 [2.95–4.31]**	**2.44 [1.64–3.66]**
**Level of family income**			
>3000€/month	418 (57.10%)	1	1
1801–3000€/month	840 (46.15%)	**0.64 [0.46–0.89]**	1.10 [0.76–1.61]
901–1800€/month	886 (37.29%)	0.97 [0.71–1.33]	1.16 [0.81–1.67]
301–900€/month	333 (27.91%)	1.32 [0.96–1.82]	1.16 [0.81–1.68]
≤300€/month	50 (40.00%)	**2.20 [1.57–3.10]**	**1.57 [1.06–2.34]**
**Internet use**			
No	312 (16.75%)	1	1
Yes	3551 (46.10%)	**4.30 [3.78–4.92]**	**1.91 [1.53–2.38]**
**Degree of disability ≥ 33%**			
No	3636 (40.39%)	1	1
Yes	224 (40.29%)	1.04 [0.87–1.23]	**1.61 [1.27–2.03]**

* Statistically significant associations are indicated in bold. ** Only statistically significant associations are shown.

**Table 4:** Person or institution informed about SH.

	*N* = 2304 *n* (%)
A family member or close friend	2113 (91.7%)
A fellow worker or student	329 (14.3%)
Reported to the police or the courts to file a complaint	96 (4.2%)
Professional service (medical, psychological, social, or legal)	79 (3.4%)
Told about it on social media (Twitter, Facebook, Instagram…)	23 (1.0%)

**Table 5:** Characteristics of women who do not talk about SH.

	Not Talk about SH *n* = 1529	Talk about SH *n* = 2335	OR [95% CI] *	*a*OR [95% CI] **
**Age**				
≥ 66	250 (16.35%)	232 (9.94%)	1	1
46–65	532 (34.79%)	677 (28.99%)	**1.45 [1.19–1.78]**	**1.43 [1.05–1.95]**
26–45	554 (36.23%)	947 (40.56%)	**1.93 [1.57–2.38]**	**1.76 [1.29–2.43]**
16–25	193 (12.62%)	479 (20.51%)	**2.78 [2.16–3.59]**	**2.34 [1.55–3.55]**
**Size of municipality**				
0–10,000	251 (16.42%)	416 (17.82%)	1	
10,001–100,000	571 (37.34%)	896 (38.37%)	1.05 [0.87–1.26]	
≥100,001	707 (46.24%)	1023 (43.81%)	1.15 [0.95–1.39]	
**Educational attainment**				
Primary or less	398 (26.03%)	806 (34.52%)	1	
Secondary (high school)	886 (57.95%)	1299 (55.63%)	**1.39 [1.20–1.62]**	
Higher (university)	244 (15.96%)	226 (9.68%)	**2.32 [1.85–2.90]**	
**Country of birth**				
Spain	1390 (90.91%)	2139 (91.61%)	1	1
Other	139 (9.09%)	196 (8.39%)	**1.29 [1.02–1.62]**	**1.46 [1.07–1.99]**
**Employment situation**				
Unemployed/Housewife	370 (24.20%)	510 (21.84%)	1	
Employed	764 (49.97%)	1242 (53.19%)	**0.82 [0.70–0.96]**	
Retired	283 (18.51%)	295 (12.63%)	**1.32 [1.07–1.64]**	
Student	107 (7.00%)	272 (11.65%)	**0.55 [0.42–0.71]**	
**Level of family income**				
≤300€/month	15 (0.98%)	35 (1.50%)	1	
301–900€/month	159 (10.40%)	174 (7.45%)	1.38 [0.80–2.41]	
901–1800€/month	366 (23.94%)	520 (22.27%)	1.11 [0.67–1.89]	
1801–3000€/month	319 (20.86%)	521 (22.31%)	0.96 [0.57–1.63]	
>3000€/month	140 (9.16%)	278 (11.91%)	0.78 [0.46–1.35]	
**Internet use**				
Yes	173 (11.31%)	139 (5.95%)	1	1
No	1356 (88.69%)	2195 (94.00%)	**2.16 [1.69–2.75]**	**1.60 [1.11–2.31]**
**Trusted person**				
Yes	1395 (91.24%)	2231 (95.55%)	1	1
No	129 (8.44%)	100 (4.28%)	**2.07 [1.56–2.77]**	**1.61 [1.12–2.33]**
**Religiosity**				
No	513 (33.55%)	1046 (44.80%)	1	1
Yes	960 (62.79%)	1199 (51.35%)	**1.55 [1.35–1.79]**	**1.26 [1.04–1.51]**

* Statistically significant associations are indicated in bold. ** Only statistically significant associations are shown.

## Data Availability

The data are available upon request from the corresponding author.

## Appendix A

**Table A1:** Forms of sexual violence included in the study.

***Partner Sexual Violence and Non-Partner Sexual Violence***		
1	You have been forced to have sexual relations by being threatened, held, or hurt in some way. By sexual relations we mean vaginal or anal penetration by the penis or by an object or objects, or oral sex.	**Rape**
2	You have been forced to have sexual relations when you were incapable of refusing because you were under the influence of alcohol or drugs.	
3	You have had sexual relations without wanting to because you were afraid of what might happen if you refused.	
4	You have been coerced into having sexual relations when you did not want to.	
5	Attempts have been made to coerce you into having sexual relations against your will, without success.	**Sexual assault/abuse**
6	Your private parts—genitals or breast(s)—have been touched or you have been touched sexually in some other way when you did not want this to happen.	
7	You have been made to touch another person’s private parts—genitals or breast(s)—or forced to touch them sexually in some other way when you did not want to do so.	
8	You have been forced to engage in some other practice of a sexual nature that has not already been mentioned.	
***Sexual Harassment***		
1	You have been subjected at some point to staring or leering that made you feel intimidated	**Intimidation**
2	Someone has shown or sent you sexually explicit images or photos that made you feel offended, humiliated or intimidated	**Exhibitionism**
3	Someone has exposed themselves to you indecently	
4	Someone has forced you to view pornographic material against your will	
5	You have received sexual jokes or offensive comments about your body or your private life	**Inappropriate content**
6	You have received inappropriate, humiliating, intimidating or offensive insinuations on online social media such as Facebook, Instagram or Twitter	
7	You have received inappropriate sexually explicit emails, WhatsApp messages or text messages that made you feel offended, humiliated or intimidated	
8	You have received inappropriate suggestions for going on a date or engaging in some activity of a sexual nature that made you feel offended, humiliated or intimidated	**Inappropriate** **suggestions**
9	You have been subjected to unwanted physical contact, such as standing unnecessarily close to you, touching parts of your body, kisses/embraces, or anything else that you did not want	**Inappropriate** **contact**
10	Someone has threatened you with unpleasant consequences in your job, such as dismissal, for example, if you rejected proposals or sexual advances	**Workplace**
11	You have experienced other similar types of behavior with sexual connotations that made you feel offended, humiliated or intimidated and that have not already been mentioned	**Other**
